# The Burden of Stroke Mimics Among Hyperacute Stroke Unit Attendees with Special Emphasis on Migraine: A 10-Year Evaluation

**DOI:** 10.7759/cureus.59700

**Published:** 2024-05-05

**Authors:** Hassan A Farid, Aaizza Naqvi

**Affiliations:** 1 Neurology, St George's University of London, London, GBR; 2 Neurology, Sheffield Teaching Hospitals, Sheffield, GBR

**Keywords:** sheffield, hyperacute stroke unit (hasu), tia mimics, migraine aura, stroke mimics

## Abstract

Background and objectives: Stroke and migraine are common neurological illnesses that cause tremendous suffering for patients. Certain diseases can mimic the clinical manifestations of an actual stroke. Migraine is one of the most commonly reported stroke mimics. The main goals of this study are to look at the prevalence of stroke mimics on the stroke pathway of Sheffield Teaching Hospitals and how many of them are migraines.

Materials and methods: A retrospective service evaluation was conducted at the hyperacute stroke unit (HASU) of the Royal Hallamshire Hospital (RHH) in the United Kingdom. The total admissions from 2013 to 2022 were collected from the Sentinel Stroke National Audit Programme database, and the number of stroke mimics was evaluated each year. The burden of migraine stroke mimics was also evaluated. Then, a one-year sample of stroke mimics was extracted to look for the types of each mimic.

Results: From 2013 to 2022, 45.75% (n = 12156) of the stroke pathway patients (n = 26573) were stroke mimics, with an increment of up to 55% in the years 2021 and 2022. During these 10 years, migraine stroke mimics accounted for 10.21% of admissions (n = 1240). The three most common mimics in a one-year sample of stroke pathway patients were migraine (14.70%) (n = 373), functional neurological disorders (FNDs) (7.17%) (n = 182), and Guillain-Barré syndrome (6.66%) (n = 169). Seizures, syncope, and metabolic derangements were reported as mimics in 4.17% (n = 106), 3.14% (n = 80), and 1.77% (n = 45), respectively.

Conclusions: About half of the HASU attendees were stroke mimics rather than actual strokes, and the most common mimics were migraines.

## Introduction

The hallmark of a stroke is the sudden onset of focal neurological impairment [1]. Since many treatments for acute stroke are time-dependent, it is important to find acute ischemic insults as rapidly as possible [2]. On the other hand, stroke overdiagnosis, formerly known as stroke mimics, may result from the pressure to make quick diagnostic and therapeutic judgments [3]. Furthermore, the problem of the wrong diagnosis in people who might have had an ischemic stroke or transient ischemic attack (TIA) is still a diagnostic challenge [4].

Stroke mimic is a descriptive term rather than a medical diagnosis. It is applied when an acute stroke has been ruled out and a more logical reason for a clinical presentation has been discovered. Depending on the clinical situation and the level of experience of those evaluating the patient, the incidence of stroke mimics might range from 10% to 30% or even more [5]. Furthermore, it is reported that the most common possible stroke mimics are seizures, syncope, infections, migraine, space-occupying lesions, functional neurological disorders (FNDs), peripheral neuropathies, and metabolic diseases [6].

Acute stroke misdiagnosis is a significant issue in healthcare, and it has serious ramifications, such as the dangers of delivering the wrong medication in an emergency situation or using the long-term stroke prevention strategy when it is not necessary. Also, the use of beds, intensive hyperacute tests, and thrombolytics have a high upfront cost, in addition to the overuse of specialist consults, including stroke/general neurologist and neurointerventionist consultations [7]. Moreover, the risk of transporting stroke mimics unnecessarily to a hospital designated for strokes can result in the inefficient use of scarce resources [8].

Regarding migraine, about 20% of the population is affected by this illness, and 20%-30% of migraines have an aura [9,10]. According to the International Classification of Headache Disorders (ICHD)-3 definition, migraine with aura is a unilateral, fully reversible visual, sensory, motor, or other symptoms that occur repeatedly last minute, usually appears gradually, and is followed by a headache and migraine-related symptoms [11]. The focal deficits associated with migraine are mostly due to aura; hence, migraine with aura accounts for roughly 9% of stroke mimics [2]. Therefore, a migraine without an aura is unlikely to imitate TIA or ischemic stroke [2,12].

In emergency settings, obtaining a history of symptoms progression can be challenging in practice because patients may report a sudden rather than gradual or spreading pattern or false lateralization, which makes it more difficult to differentiate between stroke and migraine aura clinically [13]. Aura symptoms can mimic stroke symptoms since they might be numerous, such as weakness, numbness, or speech problems. For instance, a visual and hemisensory aura together may indicate an infarction in the region of the posterior cerebral artery [14]. Additionally, “hemiplegic migraine,” or migraine aura in the form of motor weakness, may be most frequently confused with a stroke. Also, people with hemiplegic migraine usually have motor problems for up to 72 hours, but they can last for weeks [15]. Although in the hyperacute setting, it might be particularly difficult to distinguish between a migraine aura and an acute ischemic insult, evidence stated that a patient who may have had an ischemic stroke but is suitable for thrombolytic therapy should not wait for neurological deficits to recover or alter before making a diagnosis [16].

Recently, a variety of clinical scores have been validated to aid physicians in quickly and accurately distinguishing between acute stroke and its mimics. However, none of these measures were specifically created to differentiate between real cerebral ischemia and headache disorders [12]. As there is no standard method for the TIA diagnosis and there is substantial diagnostic disagreement even among experienced stroke physicians, distinguishing between migraine and TIA might be more difficult than distinguishing between stroke and migraine [17]. In fact, the most common TIA mimic may be migraine aura [18]. Inability to diagnose TIA may lead to delay in initiating secondary prophylaxis, such as dual antiplatelet medication, which can greatly lower the risk of recurrent stroke [19]. Meanwhile, TIA overdiagnosis might subject patients to costly tests, therapies that aren't essential, unnecessary driving restrictions, and insurance issues [20].

The purpose of this study is to evaluate the prevalence of stroke mimics throughout the stroke pathway and to determine how many of these mimics are migraine related.

## Materials and methods

A retrospective cross-sectional service evaluation study was conducted from June 10 to August 10, 2022, at the hyperacute stroke unit (HASU) of the Royal Hallamshire Hospital (RHH), Sheffield Teaching Hospitals (STH), and the National Health Services (NHS) Foundation Trust, Sheffield, United Kingdom. The data was recruited from the Sentinel Stroke National Audit Programme (SSNAP) database.

**Figure 1:**
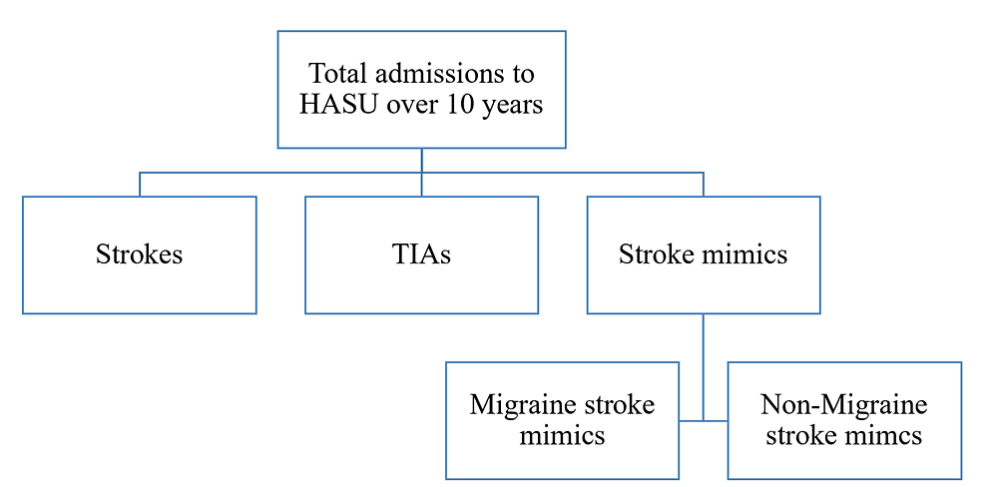
The recruitment of stroke mimics in the stroke pathway HASU: Hyperacute stroke units; TIA: transient ischemic attack

The total admissions to the HASU over a 10-year period from January 2013 to June 2022 were collected and subdivided into three groups according to the coding system for the diagnosis of "stroke," “transient ischemic attack," and “stroke mimics.” The number of patients in each group was calculated each year. Those with stroke mimics during the last 10 years were further subclassified into “migraine stroke mimics” and “non-migraine stroke mimics," and the number of patients in each group was also calculated annually (Figure 1). All admissions over these 10 years have been included, with no cases excluded. In addition, a sample of stroke mimic patients from April 2021 to June 2022 was analyzed to evaluate various types of stroke mimics.

The analysis of the study results was done using the IBM SPSS Statistics for Windows, Version 26 (Released 2019; IBM Corp., Armonk, New York, United States). The frequency and percentage represent the qualitative data. Tables, bars, and pie charts were used for data presentation. The statistical significance was assessed using the Chi-square test, with a significance level set at p < 0.05, indicating statistical significance.

## Results

**Figure 2:**
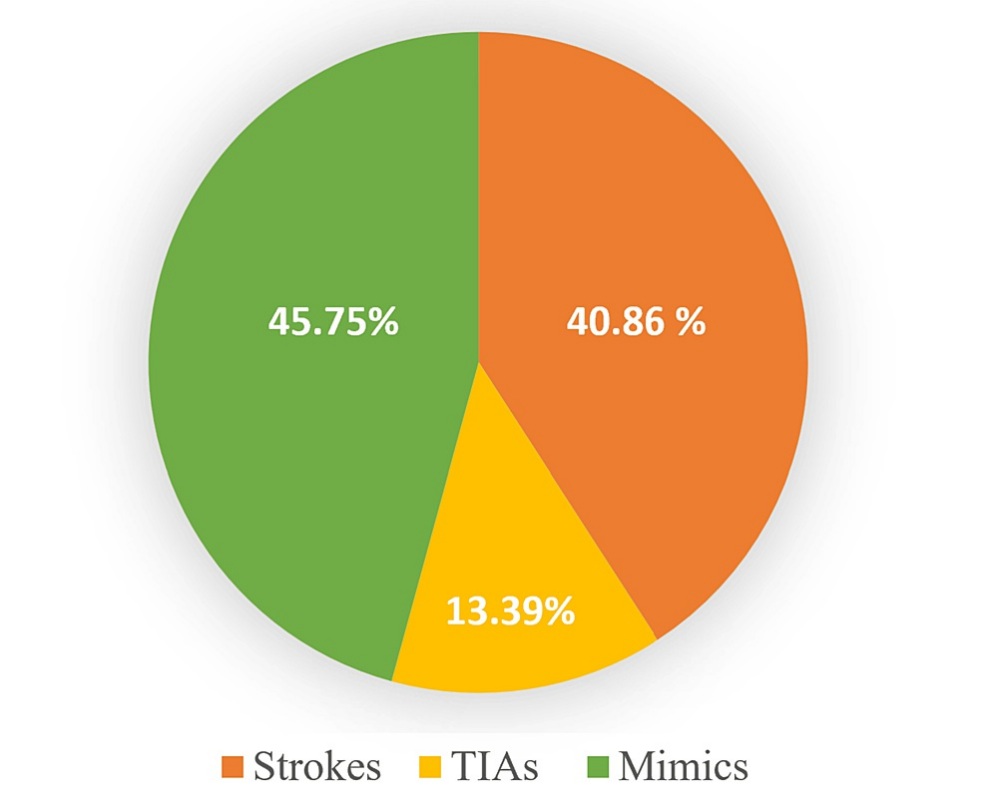
The diagnosis of all patients presented to HASU from January 2013 to June 2022 HASU: Hyperacute stroke unit; TIAs: transient ischemic attacks

The total number of admissions to the HASU from January 2013 to June 2022 was 26,573. The given diagnosis in about half of those patients (45.75%) was stroke mimics, compared to 40.86% of patients with acute stroke and 13.39% for TIAs (Figure 2).

Moreover, the distribution of cases in each year demonstrates that the number of acute stroke cases was approximately similar to the number of stroke mimics in the years from 2013 to 2020. However, the number of stroke mimics increased in 2021 and 2022 compared to the number of acute stroke cases. Noticeably, the diagnosis of TIA was also declining, especially in the last four years, and this was accompanied by an evident increase in the stroke mimics. The TIA percentage during 2022 was approximately 1/3 of that during 2013 (6.23% in 2022 vs 18.71% in 2013) (Table 1).

**Table 1:** Diagnosis of patients presented to the HASU per each year HASU: Hyperacute stroke unit; TIA: transient ischemic attack

Year	Stroke frequency (%)	TIA frequency (%)	Mimics frequency (%)	Total
2013	897 (40.64%)	413 (18.71%)	897 (40.64%)	2207
2014	939 (42.68%)	386 (17.54%)	875 (39.77%)	2200
2015	962 (41.59%)	427 (18.46%)	924 (39.95%)	2313
2016	952 (41.11%)	408 (17.62%)	956 (41.28%)	2316
2017	951 (36.77%)	589 (22.78%)	1046 (40.45%)	2586
2018	962 (40.52%)	364 (15.33%)	1048 (44.14%)	2374
2019	1171 (46.36%)	210 (8.31%)	1145 (45.33%)	2526
2020	1339 (44.41%)	243 (8.06%)	1433 (47.53%)	3015
2021	1372 (38.82%)	303 (8.45%)	1909 (53.26%)	3584
2022	1314 (38.06%)	215 (6.23%)	1923 (55.71%)	3452

**Figure 3:**
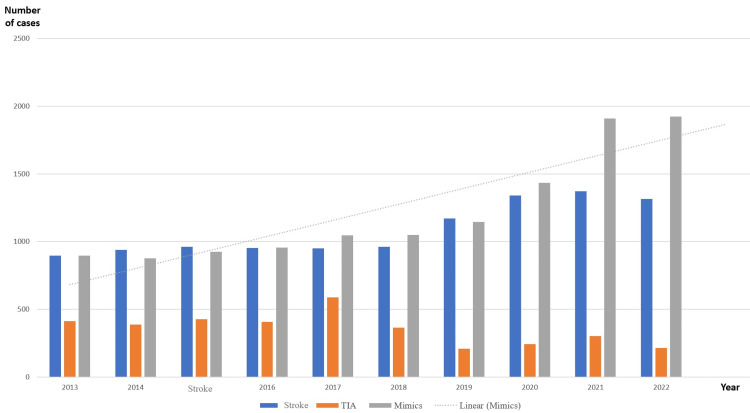
The linear increment of stroke mimics in the stroke pathway

The linear increment in the frequency of stroke mimics in comparison to acute stroke and TIA is clearly demonstrated in Figure 3.

**Figure 4:**
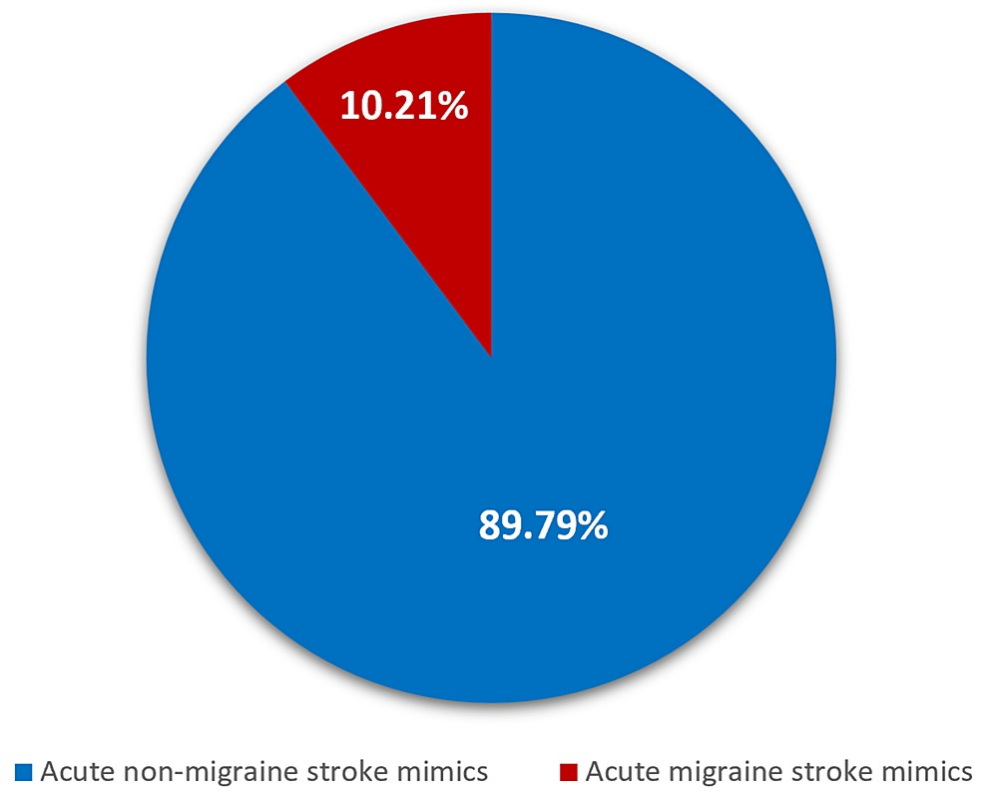
The burden of migraine stroke mimics

The subcategorization of the total number of stroke mimics which equaled to 12,156 from 2013 to 2022 into migraine stroke mimics and non-migraine stroke mimics demonstrated that 1240 (10.21%) of the cases were migraine stroke mimics in nature (Figure 4).

Furthermore, the yearly distribution of migraine stroke mimics shows that migraine was diagnosed as a mimic more frequently from 2018 to 2022 compared to 2013 to 2017 (Table 2). This difference is statistically highly significant (p < 0.01) (Table 3). 

**Table 2:** The yearly distribution of migraine and non-migraine stroke mimics HASU: Hyperacute stroke unit; TIA: transient ischemic attack

Year	Migraine stroke mimics frequency (%)	Non-migraine stroke mimics frequency (%)	Total
2013	47 (5.23%)	850 (94.77%)	897
2014	83 (9.48%)	792 (90.52%)	875
2015	61 (6.60%)	863 (93.40%)	924
2016	72 (7.53%)	884 (92.47%)	956
2017	84 (8.03%)	962 (91.97%)	1046
2018	124 (13.41%)	924 (86.59%)	1048
2019	168 (14.67%)	977 (85.33%)	1145
2020	158 (11.02%)	1275 (88.98%)	1433
2021	230 (12.04%)	1679 (87.96%)	1909
2022	213 (11.07%)	1710 (88.93%)	1923

**Table 3:** Comparison of migraine and non-migraine stroke mimics between 2013-2017 and 2018-2022 HASU: Hyperacute stroke unit; TIA: transient ischemic attack

Year	Migraine stroke mimics	Non-migraine stroke mimics	Total	p-value
2013 - 2017	347 (7.39%)	4351 (92.61%)	4698	0.001
2018 - 2022	893 (11.97%)	6565 (88.21%)	7458	
Total	1240 (10.21%)	10916 (89.79%)	12156	

**Figure 5:**
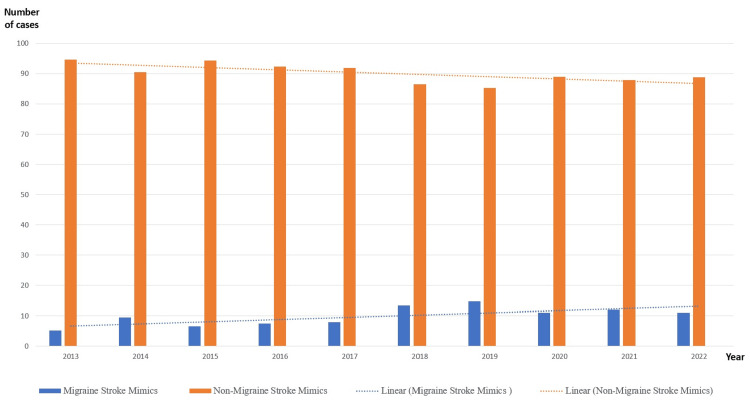
The linear increment in migraine stroke mimics

The line of increment in the percentages of migraine stroke mimics in the stroke pathway over the 10-year period is clearly shown in Figure 5.

A sample of stroke mimics patients (2536) who were presented to the stroke pathway from April 2021 to June 2022 was extracted to assess the diagnosis of the stroke mimics. The results showed that acute migraine was the most common stroke mimics (14.70%; 373), followed by FNDs (7.17%; 182) and peripheral neuropathies including Guillain-Barré syndrome (6.66%; 169), while seizure and syncope were reported as a mimic in 4.17% (106) and 3.14% (80) of the patients, respectively. Metabolic derangements including hypoglycemia were reported only in 1.77% (45) of the cases (Table 4).

**Table 4:** The diagnosis of stroke mimics

Stroke mimics diagnosis	Frequency	Percentage
Migraine	373	14.70%
Functional neurological disorders	182	7.17%
Guillain-Barré syndrome and other peripheral neuropathies	169	6.66%
Headaches excluding migraine	148	5.83%
Unknown diagnosis	144	5.67%
Spinal cord and disc lesions	135	5.32%
Facial palsy	126	4.96%
Vestibulocochlear disorders	115	4.53%
Unspecified dizziness	110	4.33%
Seizures	106	4.17%
Disorders of skin sensations	105	4.14%
Ophthalmological disorders	102	4.02%
Unspecified speech disorders	87	3.42%
Syncope and hypotension	80	3.14%
Parkinson disease and related movement disorders	60	2.36%
Systemic infections	55	2.16%
Myopathy and myasthenia gravis	52	2.05%
Anemia and fatigue	51	2.01%
Multiple sclerosis and other demyelinating diseases	48	1.89%
Nutritional deficiencies and metabolic derangements	45	1.77%
Brain tumors	41	1.61%
Undiagnosed brain disorders	37	1.45%
Alzheimer's disease and other dementia	33	1.30%
Motor neuron disease and spinal muscular atrophy	32	1.26%
Head trauma	28	1.10%
Brain infections	24	0.94%
Cerebellar ataxia	18	0.70%
Postoperative complications	12	0.46%
Gastro-intestinal swallowing disorders	10	0.39%
Hypertensive encephalopathy	4	0.15%
Drugs side effects	4	0.15%

## Discussion

Occasionally, acute ischemic stroke is difficult to diagnose. When treating a patient with a suspected acute stroke, it is essential to include stroke mimics in the differential diagnosis in order to avoid the needless use of expensive and sometimes dangerous medications. This is crucial in hospitals with a shortage of acute stroke professionals and beds [7]. It is critical to make an accurate diagnosis, concentrate on providing optimal care, use resources wisely, and avoid placing undue pressure on a highly specialized unit such as the stroke unit.

The current study revealed that the overall percentage of stroke mimics over 10 years in the RHH represented 45.75% (14256) of all admissions, ranging from 39.77% to 55.71%. This is much higher than what the other studies have stated, as a recent meta-analysis from the UK reported that only 26% of the admissions were diagnosed as stroke mimics [21]. Also, in a recent Swedish study, stroke mimics were the diagnosis in 31% of admissions [22]. However, our results were more similar to a study from a Canadian stroke center, which found that 42.6% of the patients with suspected stroke were diagnosed as stroke mimics [23]. Furthermore, according to Buck et al.'s review, the frequency of stroke mimics varies depending on where the diagnosis is made and whether the patients are examined by emergency staff or stroke specialists, and they stated that stroke mimics account for 20%-50% of acute suspected stroke cases [7]. The possible explanation for the high percentage of stroke mimics in the HASU of RHH is that the initial evaluation and referral were done by the ambulance team in the majority of admissions, and the stroke team evaluated the patients only after admission to the HASU. Moreover, there is no emergency unit in RHH and no strict referral criteria. Also, the RHH accepts stroke referrals from many centers in South Yorkshire, and this contributes to a much higher burden of stroke mimics than the other UK centers. It is crucial to acknowledge that there may be changes between the first contact and admission, which might lead to the remission of symptoms. This emphasizes the ever-changing character of stroke diagnosis and the need for prompt and precise evaluations. When it comes to staff training, the differences in the degrees of training across various staff members might be seen as an area that needs improvement. To improve the accuracy of assessments and decrease the occurrence of stroke mimics, it is important to address gaps in training levels and provide comprehensive education on stroke assessment and diagnosis.

For patients with suspected strokes, emergency medical dispatch services are frequently the initial point of contact. Often, the caller uses ambiguous, nonspecific, or maybe distracting terms that make it challenging for the dispatcher to understand the diagnosis [24]. A more recent comprehensive study revealed that just 21% of diagnoses were accurate after medical dispatchers activated an ambulance and ambulance paramedics still cannot recognize acute strokes, and too many people are sent to stroke centers when they do not need to be [23]. In order to enhance the diagnosis of acute stroke, several prehospital measures have been developed and are increasingly being used by paramedics such as the face, arms, speech, and time (FAST) scale, Recognition of Stroke in the Emergency Room (ROSIER) scale, and Los Angeles Motor Scale (LAMS); however, the studies found that all of these scales were not very accurate and missed about 30% of acute strokes [25]. Still, we found that the FABS scoring system is a validated technique specifically intended for screening stroke mimics in the emergency department. The scoring system consists of many essential elements, each of which is awarded a certain amount of points depending on whether they are present or absent in the patient's clinical presentation. The combined score derived from these components is directly related to the likelihood of stroke mimics, ranging from 0.00 to 1.00. The scoring procedure takes into account many factors, including the lack of facial drop, age below 50 years, absence of atrial fibrillation, systolic blood pressure less than 150 mm Hg, existence of isolated sensory loss, and history of seizure conditions. This systematic methodology allows doctors to accurately measure the probability of stroke mimics with a high level of sensitivity and specificity, hence aiding precise diagnostic decision-making and optimizing patient care options. The FABS score is significant because it can quantitatively evaluate the probability of stroke mimics. A higher FABS score suggests a higher likelihood of stroke mimics, with sensitivity and specificity rates of 90% and 91%, respectively, when the score is three or above [26].

It is also essential to highlight the increment in the percentage of stroke mimics during the last two years, as it reached up to 55%. Such things in turn cause a further burden on the doctors and medical staff, especially as this was coexisting with the COVID-19 pandemic, which further led to staff shortage due to obligatory sick leaves.

In the acute setting, it can be particularly difficult to discriminate between an attack of migraine aura and acute cerebral ischemia [16]. Moreover, there is no gold-standard test for the diagnosis of TIA, and there is significant diagnostic disagreement even among experienced doctors. This makes the distinction between TIA and migraine, especially that with aura, more challenging than the distinction between migraine and stroke [12,17].

Our study revealed an overall percentage of migraine, most probably with aura, equaled to 10.21%, with a range from 5%-14%, but a previous systematic analysis found a slightly higher percentage as migraine with aura was the final diagnosis in almost 18% of stroke mimics [27]. However, in the last five years in the HASU of RHH, there was noticeable decrement in the number of TIA cases with a corresponding increase in the number of migraine stroke mimics diagnoses, which may indicate that physicians are becoming more aware of the prevalence of migraine diagnosis as TIA mimics. Although there have not been any revisions in the national criteria for diagnosing stroke and stroke mimics, there has been a focused effort to improve awareness and training programs for frontline personnel.

Our evaluation reported that the top three stroke mimics were migraine (373; 14.70%), FND (182; 7.17%), and peripheral neuropathies (6.66%; 169). These results have some similarities to a report by Neves Briard et al., in 2018, who described that seizures (19.7%), migraines (18.8%), FNDs (11.9%), and peripheral neuropathies (11.2%) were the most frequent neurological mimics among a sample of 950 patients [23]. However, seizures in our evaluation represented only about 4% of the mimics, which is also far from what McClelland et al. have reported as they found that seizure was the most common stroke mimic [21]. Although Neves Briard et al. found that 15.9% of the mimics were cardiovascular-related [23], our study found that only around 3% of mimics have cardiovascular etiologies such as syncope or hypotension. 

If a patient has a suspected acute stroke and is within the thrombolysis window, they have to be assessed quickly in the emergency room for a quick diagnosis and thereby shorten the "door-to-needle" time which should be equal to less than 30 minutes [28]. However, this extremely quick evaluation often makes it harder to differentiate stroke from stroke mimics. Stroke thrombolytic therapy such as alteplase carries a significant risk of adverse effects especially intracranial bleeding [29]. Keselman et al. stated that migraine, FNDs, and seizures were the most common mimics treated by thrombolytic therapy [29].

In our study, only three patients (0.1%) out of 2536 stroke mimics received thrombolytic therapy, which was remarkably lower than the 3.5%-4.1% reported by the abovementioned study [29]. This might reflect a high level of diagnostic maturity and reliability among RHH physicians in determining those who require thrombolytic therapy. In most cases, a thorough clinical history, targeted clinical examination, and a multimodal CT scan are enough to make a diagnosis. The doctor should be aware of the possibility of a stroke mimic while reviewing the patient and should make the diagnosis carefully based on meticulous clinical judgement.

Introducing a phone or video communication prior to admission with the stroke specialist seems promising in dealing with stroke mimics [30]. In RHH, a trial of using video calls in dealing with stroke patients is currently under way, and this method is still under evaluation. It would probably have a potential benefit by reducing the number of stroke mimics.

One limitation of this research is that it relies on a coding system for data collection. This coding system may not capture all the necessary information. In addition, there may have been inconsistent recording of information on stroke mimics in the stroke route database, which might result in inadequate data. One further constraint is the study's retrospective nature, which could potentially create biases or limits in the data gathering and processing. Moreover, the research only examined a solitary HASU in the United Kingdom, thus restricting the applicability of the results to other healthcare settings or geographies.

## Conclusions

About half of the HASU attendees were stroke mimics rather than actual strokes, especially in the last couple of years, and the most common mimics were migraines. It is good to recommend the addition of a filtration step prior to admission to the HASU and assess patients initially by the stroke team to minimize the number of mimics as much as possible to reduce the cost and staff shortage burden and improve the quality of care. This might also be achieved by encouraging the use of certain scores, such as the FABS score, as it has 90% sensitivity and 91% specificity for the diagnosis of stroke mimics if it is higher or equal to three. 
